# Novel technique of strain assessment utilizing feature tracking in nontagged SSFP images: validation with tagged strain analysis

**DOI:** 10.1186/1532-429X-11-S1-P33

**Published:** 2009-01-28

**Authors:** Kan N Hor, Erin Wash, Robert J Fleck, Janaka P Wansapura, James F Cnota, D Woodrow Benson, William M Gottliebson, Wojciech Mazur

**Affiliations:** 1 CCHMC, Cincinnati, OH USA; 2 Ohio Heart and Vascular Center, Christ Hospital, Cincinnati, OH USA

**Keywords:** Duchenne Muscular Dystrophy, Duchenne Muscular Dystrophy, Feature Tracking, Duchenne Muscular Dystrophy Patient, Normal Ejection Fraction

## Introduction

Recent cardiac MRI (CMR) studies have demonstrated decline in left ventricular peak circumferential strain (ε_cc_) despite normal ejection fraction in Duchenne muscular dystrophy (DMD) patients. However, these analyses used CMR tagging, a technique limited by tag fading and complicated analysis requirements. Feature tracking software has recently been developed for analysis of ε_cc_ from non-tagged standard steady-state free precession (SSFP) cine CMR images.

## Purpose

The purpose of this study was to compare Mid-LV slice ε_cc_ by feature tracking of SSFP cine CMR images to HARP analysis of tagged images.

## Methods

ε_cc_ was assessed from CMR SSFP short-axis cine stack images and cine myocardial tagged image data of 54 DMD patients and 6 aged-matched control subjects, utilizing both tagged analysis (via HARP^®^ software, Diagnosoft Inc) and SSFP feature tracking (via DIOGENES^®^ software, TomTec Inc) methods. Analyses were performed on identical location mid-papillary LV slices (both tagged and standard SSFP). Average ε_cc_ was tabulated and compared via Spearmen rank correlation and Bland-Altman comparison of methods.

## Results

**Figure 1:**
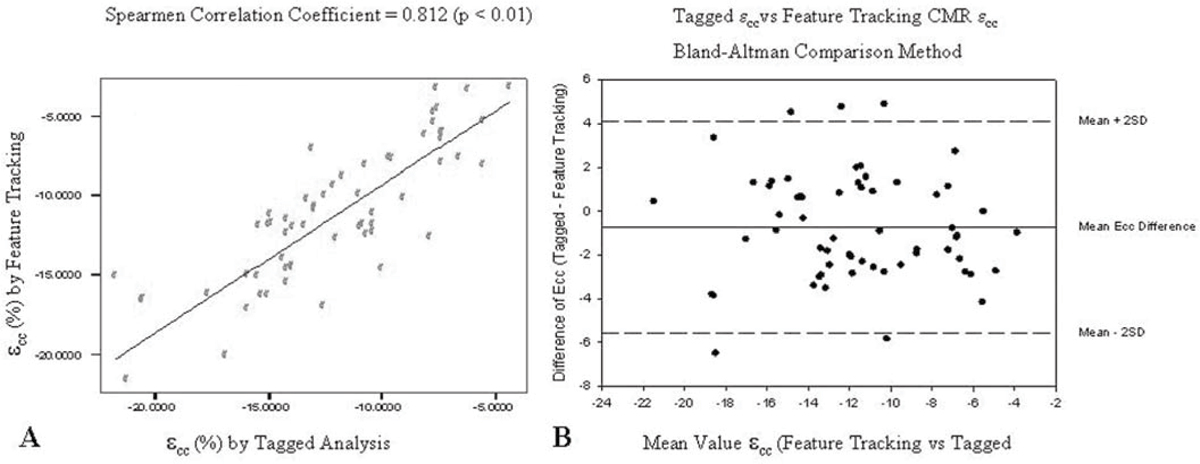
Figure 1

Feature tracking ε_cc_ analysis correlated favorably with tagged HARP analysis (fig 1a). In addition, the techniques do not demonstrate systematic over or underestimation of one another, though the limits of agreement are relatively wide (fig 1b).

## Conclusion

CMR feature tracking is a feasible method for assessment of ε_cc_. Further study on larger subject groups is warranted to determine efficacy and accuracy of feature tracking versus tagged analysis.

